# Genomic landscape of early-stage prostate adenocarcinoma in Mexican patients: an exploratory study

**DOI:** 10.1007/s12672-024-01199-3

**Published:** 2024-08-28

**Authors:** Dennis Cerrato-Izaguirre, Jonathan González-Ruíz, José Diaz-Chavez, Andrea Ramírez, Anna Scavuzzo, Miguel A. Jimenez, Carlo Cortés-González, Jairo A. Rubio, María D. Pérez-Montiel, Claudia M. García-Cuellar, Luis A. Herrera, Yesennia Sánchez-Pérez, Felipe Vaca-Paniagua, Salim Barquet-Muñoz, David Cantu-de-Leon, Promita Bose, Diddier Prada

**Affiliations:** 1 Subdirección de Investigación Básica, Instituto Nacional de Cancerología, Mexico City, Mexico; 2 Unidad de Apoyo Molecular para la Investigación Clínica, Instituto Nacional de Cancerología, Mexico City, Mexico; 3 Departamento de Urología, Instituto Nacional de Cancerología, Mexico City, Mexico; 4 Departamento de Patología, Instituto Nacional de Cancerología, Mexico City, Mexico; 5 Dirección de Investigación, Instituto Nacional de Cancerología, Mexico City, Mexico; 6 Laboratorio Nacional en Salud, Diagnóstico Molecular y Efecto Ambiental en Enfermedades Crónico-Degenerativas, Facultad de Estudios Superiores Iztacala, UNAM, Tlalnepantla, Mexico; 7 Department of Environmental Health Sciences, Mailman School of Public Health Columbia University, New York, NY USA; 8 Institute for Health Equity Research, Icahn School of Medicine at Mount Sinai, New York City, NY USA; 9 Department of Population Health Science and Policy, Icahn School of Medicine at Mount Sinai, New York City, NY USA; 10 Department of Environmental Medicine and Climate Science, Icahn School of Medicine at Mount Sinai, New York City, NY USA

**Keywords:** Prostate cancer, Health disparities, Mexican population, Mutations, Cancer genomics

## Abstract

**Background:**

Health disparities have been highlighted among patient with prostate adenocarcinoma (PRAD) due to ethnicity. Mexican men present a more aggressive disease than other patients resulting in less favorable treatment outcome. We aimed to identify the mutational landscape which could help to reduce the health disparities among minority groups and generate the first genomics exploratory study of PRAD in Mexican patients.

**Methods:**

Paraffin-embedded formalin-fixed tumoral tissue from 20 Mexican patients with early-stage PRAD treated at The Instituto Nacional de Cancerología, Mexico City from 2017 to 2019 were analyzed. Tumoral DNA was prepared for whole exome sequencing, the resulting files were mapped against h19 using BWA-MEM. Strelka2 and Lancet packages were used to identify single nucleotide variants (SNV) and insertions or deletions. FACETS was used to determine somatic copy number alterations (SCNA). Cancer Genome Interpreter web interface was used to determine the clinical relevance of variants.

**Results:**

Patients were in an early clinical stage and had a mean age of 59.55 years (standard deviation [SD]: 7.1 years) with 90% of them having a Gleason Score of 7. Follow-up time was 48.50 months (SD: 32.77) with recurrences and progression in 30% and 15% of the patients, respectively. *NUP98* (20%), *CSMD3* (15%) and *FAT1* (15%) were the genes most frequently affected by SNV; *ARAF* (75%) and *ZNF419* (70%) were the most frequently affected by losses and gains SNCA’s. One quarter of the patients had mutations useful as biomarkers for the use of PARP inhibitors, they comprise mutations in *BRCA*, *RAD54L* and *ATM*. SBS05, DBS03 and ID08 were the most common mutational signatures present in this cohort. No associations with recurrence or progression were identified.

**Conclusions:**

This pilot study reveals the mutational landscape of early-stage prostate adenocarcinoma in Mexican men, providing a first approach to understand the mutational patterns and actionable mutations in early prostate cancer can inform personalized treatment approaches and reduce the underrepresentation in genomic cancer studies.

**Supplementary Information:**

The online version contains supplementary material available at 10.1007/s12672-024-01199-3.

## Introduction

Prostate adenocarcinoma (PRAD) stands as the most prevalent cancer among men on a global scale and represents 95% of all the prostate cancers. An estimated of 1,414,259 new cases are diagnosed yearly in a global basis, in Mexico around 26,742 new cases were estimated in 2020, making PRAD by far the most diagnosed cancer in Mexican male patients, above colorectal cancer [1]. Despite recent advancements, PRAD remains a significant medical challenge for individuals affected by the disease [2]. The prevalence of PRAD rises markedly with increasing age [3].

Health disparities have been highlighted among patients in the United States affecting especially racial and ethnic minority groups like Hispanic patients. These health disparities along the continuum of care for PRAD management have a negative impact on the clinical outcomes [4]. Hispanic men are often diagnosed with prostate cancer at more advanced stages of the disease compared to non-Hispanic white men. Late diagnosis can result in less favorable treatment outcomes [5]. In Mexico, a low survival rate has been found in residents of highly marginalized municipalities, due to the lack of social services, low education levels and low wage incomes [6]. Additionally, variances in incidence among different racial and ethnic group exist [2], these differences in incidence could be due to genetic influence [7].

Racial differences in genomic profiling of patients with PRAD have been reported. For example, Black men are more likely to present DNA repair mutations and androgen receptor mutations than Asian and White men [8]. Also, Black men with metastatic disease are more likely to have actionable mutations [8]. On the other hand, Asian men are more likely to harbor *TP53* and *FOXA1* mutations in the primary tumor [8]. However, very little genetic information can be found about Hispanics patients. The first description of the frequency of DNA alterations in primary and metastatic prostate cancer of Hispanic men, published in 2023, identified a higher frequency of *TMPRSS2*, *ERG*, and *PPARG* alterations in Hispanic men than non-Hispanic men but no differences in the prevalence of actionable genetic alterations were found between Hispanic and non-Hispanic patients [9]. Among the Hispanic community, Mexican men present a more aggressive disease with an advanced stage at diagnosis and mortality rates are higher compared to other Hispanic American and No-Hispanic White men [10]. The Latino population, characterized by genetic admixture from various ancestral populations [11], however, genetic alterations in Hispanic men with PRAD are less described than other ethnic groups [12].

The identification of the mutational alterations underlying the PRAD biology presented by underrepresented minorities such as Hispanic men could help diminish the representational gap between the diverse racial and ethnic groups, particularly in the early stage. Conducting genetic studies in the early stages of prostate cancer could improve patient care by generating genomic information that could lead to a better diagnosis or a personalized treatment. Here, we aimed to identify the mutational landscape, the actionable genetic mutations, and the mutational processes related to Mexican men with PRAD with an early-stage disease, which could help to reduce the health disparities among minority groups and generate the first genomics exploratory study of PRAD in Mexican patients.

## Materials and methods

### Population

We randomly selected 20 patients, treated at the Instituto Nacional de Cancerología (INCan) in Mexico City between 2017 and 2019 from a pool of eligible patients. To be eligible, patients were required to have formalin-fixed paraffin-embedded (FFPE) tumor tissue blocks stored within the pathology department of INCan, to be 18 years old or older, and to have a confirmed diagnosis of early-stage PRAD. FFPE samples were collected prior to any form of therapeutic intervention. Individuals with a history of other malignancies or treatment were excluded from the study. The selection of tumor samples was meticulously done by an experimented oncologist pathologist (Dr. Perez-Montiel) in the formalin-fixed FFPE blocks. Notably, the chosen tumor samples exhibited a minimum of 70% tumor cellularity.

### Clinical data collection

Essential clinical data including age at diagnosis, gender, level of education, smoking status, and relevant symptoms upon diagnosis, were extracted from the electronic clinical records pertaining to each individual patient. The duration of follow-up was calculated by referencing the clinical records, which defined the interval between the initial diagnosis date and either the date of mortality or the loss of follow-up.

### DNA extraction and quality control

Samples were homogenized using QIAshredder (QIAGEN, 79654), followed by DNA extraction using the QIAamp^®^ DNA FFPE Tissue Kit (QIAGEN, 56404), in adherence to the recommended protocol. The DNA's purity was assessed using the Thermo Fisher Scientific NanoDrop 2000. Additionally, the quantity of DNA and its fragmentation status were evaluated utilizing the Agilent 2200 TapeStation System, employing the genomic DNA ScreenTape assay (Agilent, 5067–5365), which facilitated the calculation of the DNA integrity number [13]. Samples featuring DNA integrity numbers within the range of 6–10, along with a minimum concentration of 20 ng/μl, were selected as suitable candidates for whole-exome sequencing (WES).

### Library preparation, hybridization capture, and WES

Library preparation, hybridization capture, and the subsequent WES processes were performed by the New York Genome Center. TruSeq DNA PRADR-Free libraries were prepared using 1 μg of input DNA sourced from FFPE tissues, following the manufacturer's guidelines (Illumina, San Diego, CA, USA). The sequencing phase was conducted on the HiSeq2500 platform (Illumina, San Diego, CA, USA) in Azenta, NY, USA (https://www.azenta.com/).

### Bioinformatics pipeline

Sequencing reads originating from the tumor samples underwent preliminary adapter trimming through Trim-Galore (v0.4.0). Subsequently, these trimmed reads were aligned to the reference genome via BWA-MEM (v0.7.15) [14]. Further processing included the utilization of GATK (v4.1.0) [15] for fixing and verifying mate-pair information through the execution of FixMate Information. The consolidation of individual lane BAM files into a unified BAM file per sample was achieved using Novosort (v1.03.01) markDuplicates [16]. Post-duplicate handling, sorting, and marking ensued, followed by the implementation of GATK’s base quality score recalibration to yield a coherent, sorted BAM file for each sample. Given the unavailability of a matched normal sample, HapMap sample NA12878 was employed as a surrogate. This substitute normal sample, prepared and sequenced following an identical protocol to the tumor sample, was utilized to eliminate spurious positives stemming from library preparation and sequencing, shared between the tumor and NA12878. It also facilitated the removal of certain germline variants shared between the tumor sample and NA12878. The tumor and normal BAM files were processed using GATK (v4.0.5.1) [17], Strelka2 (v2.9.3) [18] and Lancet (v1.0.7) [19] for calling single nucleotide variants (SNV’s) and small insertions and deletions (InDels), SvABA (v0.2.1) [20] for calling InDels, and FACETS (v0.5.5) [21] for calling somatic copy number alterations (SCNA’s). High-confidence variants identified by at least two variant callers and variants with a variant allele frequency equal to or in between 0.1 and 0.45 were selected for subsequent analysis. SNVs and Indels were annotated with Ensembl, as well as databases such as COSMIC (v86) [16] 1000Genomes (Phase3) [22], ClinVar (201706) [23], PolyPhen (v2.2.2) [14], SIFT (v5.2.2), FATHMM (v2.1), [24] gnomAD (r2.0.1) [25] and dbSNP (v150) [26] using Variant Effect Predictor (v93.2) [27]. Synonymous mutations and mutations annotated in non-coding regions were filtered out.

Regarding the SCNA’s, segments demonstrating log2 values exceeding 0.2 were classified as amplifications, while those with log2 values below -0.235 were classified as deletions. This threshold corresponded to a single copy change at 30% purity within a diploid genome or a 15% variant allele fraction. SCNA’s with a size less than 20 Mb were categorized as focal, whereas larger SCNA’s were considered large-scale. Only focal SCNA’s were selected for subsequent analysis. Bed tools was utilized for SNCA annotation. Furthermore, all predicted SCNA’s were annotated by overlapping with known germline variants sourced from 1000 Genomes and the Database of Genomic Variants (DGV) [28].

### Mutational signature analysis

An analysis of mutational signatures for single-base substitutions (SBS) was performed to elucidate the distinct roles of various mutational processes in the context of carcinogenesis, according to COSMIC database as a guiding reference (REF). Sigprofiler Assignment web tool was used to identify SBS, double base substitutions (DBS), and insertion and deletion (ID) signatures (https://cancer.sanger.ac.uk/signatures/assignment/) [29].

### Actionable genetic mutations

The biological and clinical relevance of SNVs and InDels was identified using the Cancer Genome Interpreter (CGI) web interphase (https://www.cancergenomeinterpreter.org/home) [30]. To predict driver mutations, a tissue-specific model for PRAD was selected. CGI uses a machine learning algorithm named BoostDM to annotate the clinical and biological relevance of the somatic variants with a BoostDM score of 0.5 and an accuracy for predicting driver mutations (F50-Score) above 0.9 [31].

### Statistical analysis

This is a descriptive pilot study where clinical features were described according to the genomic variants and mutational landscape of patients and no statistical associations were assessed due to limited sample size.

## Results

### Clinical-pathological characteristics

The mean age of patients was 59.55 years (standard deviation [SD]: 7.1 years) with a mean time to follow-up of 48.50 months (SD 32.77). Of the total patients, 30% had an educational level of high school or college, and 50% had a history of smoking and alcoholism. Nearly all the patients had a 7 Gleason score (90%), and all the patients were on an early clinical stage, both at diagnosis clinical stage at diagnosis and at the time of sample collection. More details of the characteristics of the patients are shown in Table 1.

**Table 1:** Demographic characteristics of patients with prostate cancer treated at the Instituto Nacional de Cancerología between 2017 and 2019 (N = 20)

Variable	Mean	SD
Age (years)	59.55	7.1
Follow on time (months)	48.50	32.77

	N	%
Education		
High school or lees	9	45%
College or vocational school	5	25%
Graduate school or higher	6	30%
Smoking		
Yes	10	50%
Alcohol consumption^a^		
Yes	11	55%
Symptoms at diagnosis		
Asymptomatic	11	55%
Symptomatic^a^	7	35%
Hematuria	2	10%
Diabetes mellitus type 2		
Yes	4	20%
Blood hypertension		
Yes	7	35%
PSA at dx	12.179	9.89
Gleason score		
7	18	90%
8	1	5%
9	1	5%
Clinical stage		
II	2	10%
IIA	1	5%
IIB	1	5%
IIC	13	65%
Radiotherapy		
Yes	9	45%
Recurrence		
Yes	6	30%
Progression		
Yes	3	15%

*SD* standard deviation, *PSA* prostate-specific antigen

^a^Irritative symptoms during urination (dysuria or burning sensation)

^b^Based on clinical records

### Mutational landscape

**Fig. 1:**
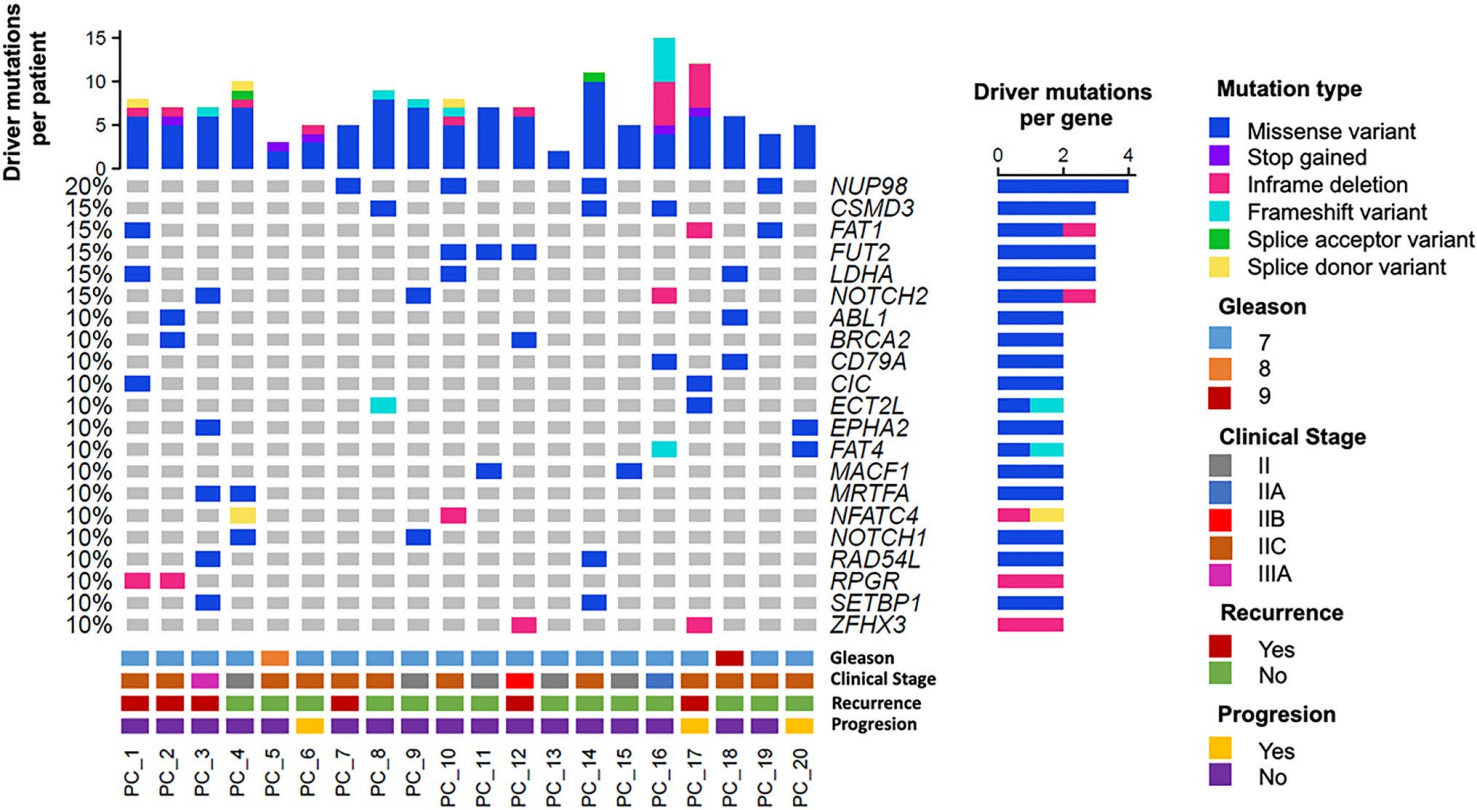
Mutational landscape and clinical features of Mexican patients with early-stage prostate adenocarcinoma. Oncoprint depicting the genes with driver mutations found in a cohort of 20 Mexican patients with early-stage prostate adenocarcinoma. The upper bar graph represents the driver mutations found per patient. The right bar graph represents the frequency of driver mutations found per gene. Mutation type, Gleason score, clinical stage, recurrence, and progression per each patient is represented according to the color code of the legend panel

WES of the FFPE samples patients with early-stage PRAD was achieved successfully with a mean sequencing depth of 182.90X and a target region coverage of 99.65%. A total of 30,904 somatic variants were identified with a mean of 7062.35 mutations (SD: 1963.62 mutations) per sample. After grouping driver and passenger mutations, 145 driver mutations were found affecting 116 different genes. Missense variants were the most common mutation type, followed by in frame deletions. The nucleoporin coding gene *NUP98* was the most frequently affected in 20%of the patients, always with the p.R964C mutations. All patients with *NUP98* mutations were in an early clinical stage (IIA), and did not present progression or recurrence, except one. This patient consumed alcohol, a smoker and, was treated with radiotherapy. *CSMD3* (15%), *FAT1* (15%), *FUT2* (15%), *LDHA* (15%) and *NOTCH2* (15%) were also genes mutated in this cohort (Fig. 1).

**Fig. 2:**
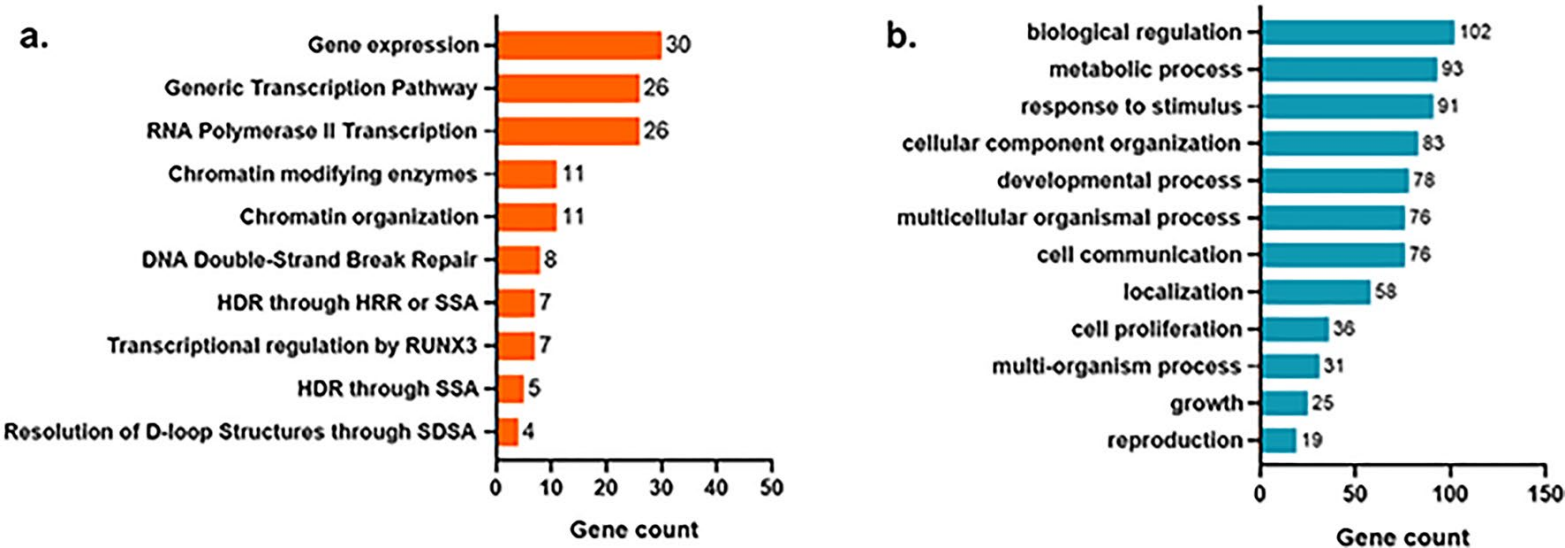
Molecular pathways affected by driver somatic variants from patients with early-stage prostate adenocarcinoma. **a** Bar plot representing the molecular pathways affected by driver somatic variants using Reactome as reference dataset. **b** Bar plot representing depicting the biological processes affected by driver somatic variants, using as reference the Gene Ontology dataset

To identify the signaling pathways affected by driver mutations we performed an enrichment analysis using the overrepresentation tools of Reactome (https://reactome.org). Gene transcription related pathways were the most affected by driver mutations (Fig. 2a). *NUP98* was present only in the Reactome geneSet R-HSA-74160, corresponding to Gene expression. The Resolution of D-loop Structures through Synthesis-Dependent Strand Annealing as the most strongly enriched molecular pathway (Enrichment Ratio [ER]: 18.45, *p*-value = 0.013) with 4 genes, *ATM, BRCA2, RAD50* and *WRN.* Additionally, we identified the biological processes affected using Gene Ontology, where the pathway with more affected genes was the Biological Regulation pathway GO:0065007. Followed by metabolic process (GO:0008152), response to stimulus (GO:0050896) and the cellular component organization (GO:0016043) (Fig. 2b).

### Actionable genetic mutations

**Fig. 3:**
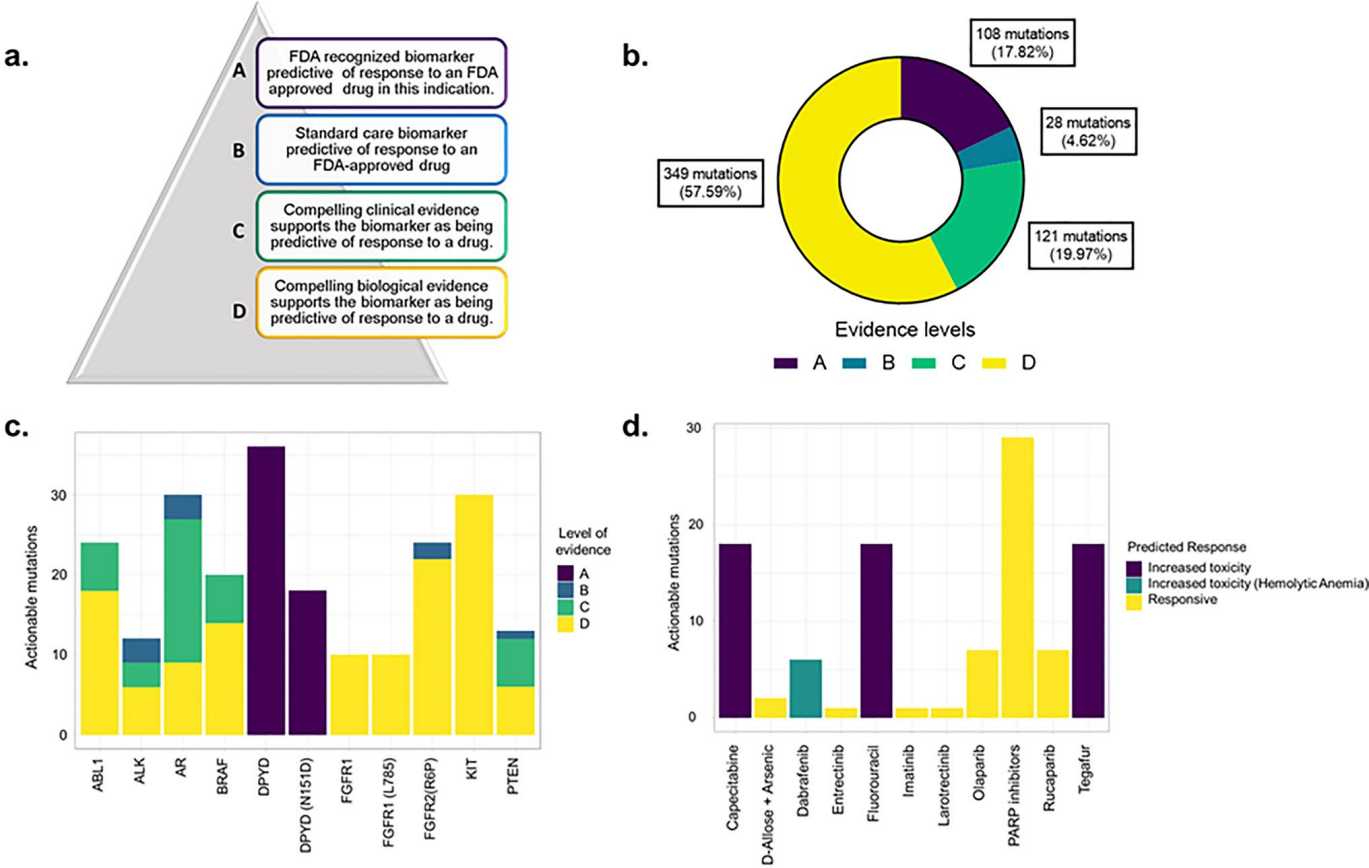
Actionable genetic mutations identified in early-stage prostate adenocarcinoma. **a** evidence levels of the predicted biomarker function of the actionable mutations. **b** Frequency of actionable mutations according to the evidence levels. **c** Genes with high frequency of actionable mutations grouped according to evidence levels. **d** Drugs with predicted response related to actionable mutations

We evaluated the clinical utility of the actionable mutations identified in this cohort using the CGI. We identified a total of 5058 actionable mutations among the 20 patients. However, after filtering only for the mutations associated with PRAD or “Any Cancer type”, 606 were identified. The predicted response of those actionable mutations to chemotherapeutic agents was classified according to the evidence levels presented in Fig. 3a. Most of the actionable mutations (57.59%) had a D evidence level (Fig. 3b). However, the encoding gene to dihydropyrimidine dehydrogenase, *DPYD*; was the gene with the greatest number of actionable mutations and all of them had an A evidence level (Fig. 3c). An increment of toxicity to Capecitabine based treatment was predicted due to intronic and exonic mutations in *DPYD* in six patients (PC_6, PC_8, PC_13, PC_14, PC_18, and PC_20) and increased toxicity to Fluorouracil treatment was also predicted due to *DPYD* mutations (Fig. 3d). We also identified driver mutations related to PARP inhibitors response in two patients harboring *BRCA2* mutations (p.A2952T), another two patients harboring *RAD54L* mutations (p.P492L, p.R202C) and one patient with an *ATM* (p.H2872X) mutation, accounting for a total of 5 patients (25%) that could be candidates for the use of Poly [ADP-ribose] polymerase (PARP) inhibitors.

### Somatic copy number alteration

We also explored the SCNA’s present in patients with early-stage PRAD and identified a total of 636 SCNA among the 20 patients. We filtered for those SNCA’s that were present in COSMIC, Cancer gene census, and DGV databases, resulting in 485 SCNA’s. When exploring only focal SCNA’s, deletions in 5q31.3 were observed in all the patients, involving the genes *ZMAT2* and *PCDHA1* (Fig. 3a). Other cytobands frequently affected included 19q13.42 (73.68%), 19q13.2-q13.31 (68.42%), 14q11.2 (63.15), 14q32.33 (52.63%), 2q14.3-q21.2 (52.63%), 2q31.2 (52.63%), and 7q22.1 (52.63%).

**Fig. 4:**
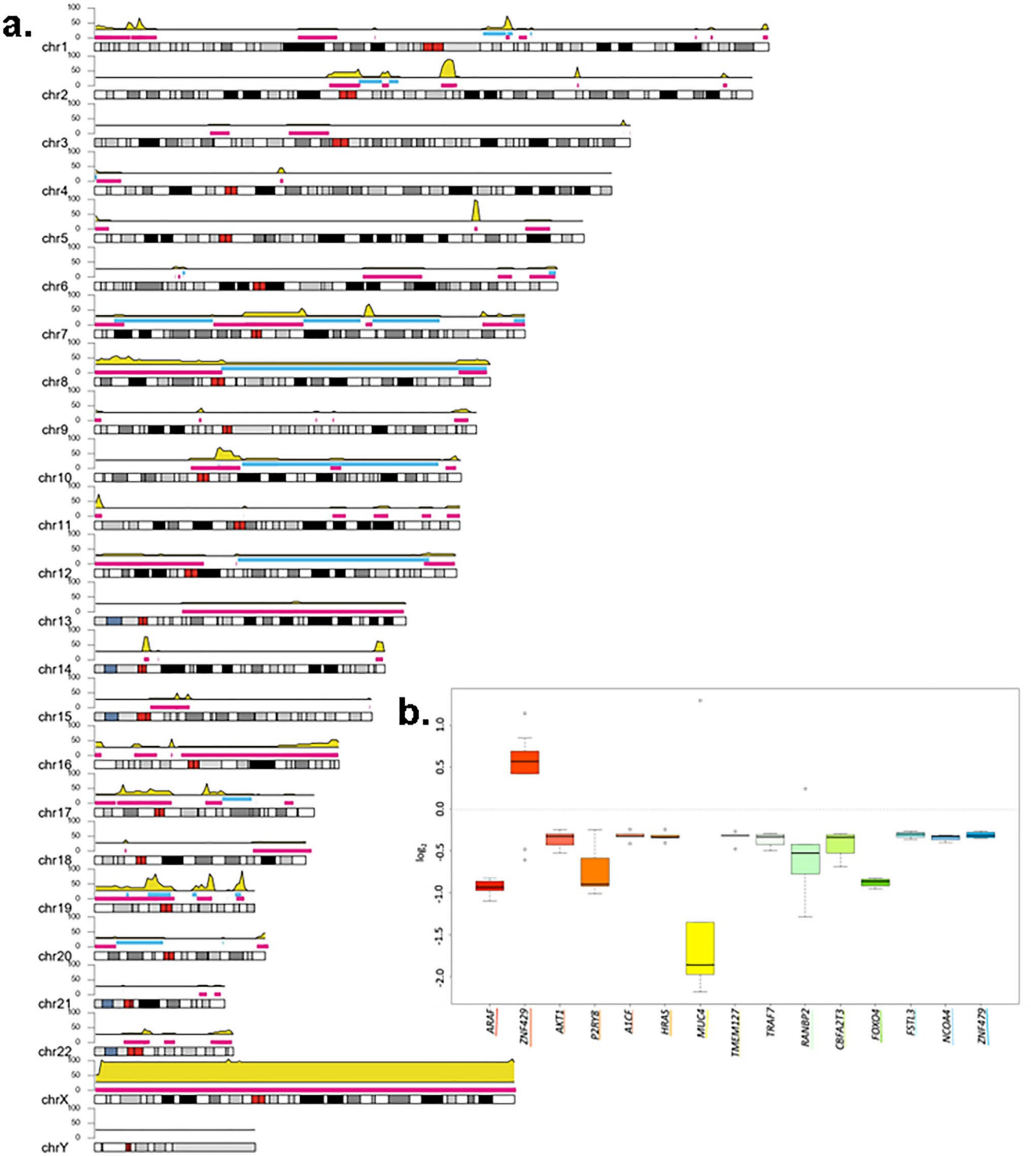
Somatic copy number alterations (SCNA’s) present in early-stage prostate adenocarcinoma (N = 20). **a** Karyoplot depicting the genomic regions affected by SCNA’s in all the patients (n = 20). Blue represents the gains, pink, the losses. The yellow density plot represents the frequency of patients affected by SCNA’s across the genome. Top-ten frequently affected cytobands across all samples are shown in a box in bold. **b** box plot showing the log2 values of 15 most frequently affected genes, if the values exceeding 0.2 were classified as gains, while those values below -0.235 were classified as losses

We observed a total of 29 gains and 167 losses in genes affected by SCNAs across the 20 patients, with a mean of 2.9 gains (median = 1.5) and 8.78 losses (median = 5) per sample. In Fig. 4a, we can observe the Karyoplot depicting the genomic regions affected by SCNA’s in all the patients. Large deletions in Xp22.33-q28 affecting *ARAF* were presented in 75% of the patients (mean log2 = − 0.944, SD: 0.065). *ZNF429, AKT1, P2RY8,* and *A1CF* were the following more frequent genes affected by SCNA’s (Fig. 4b).

### Mutational profile and mutational processes associated with prostate *cancer*

**Fig. 5:**
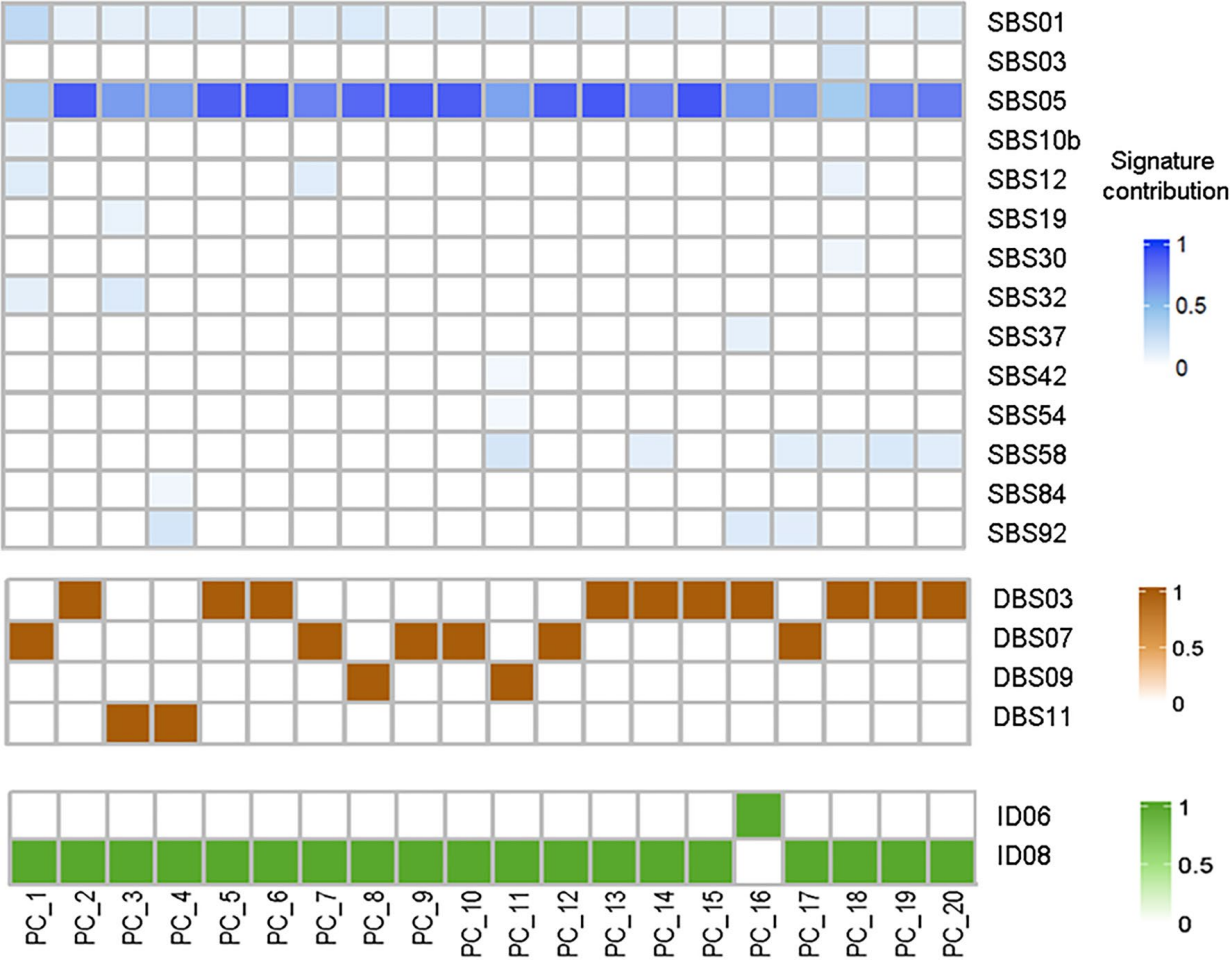
Mutation processes of Mexican patients with early-stage prostate adenocarcinoma. Blue table represents single substitution signatures (SBS), brown table represents double base substitution signatures (DBS) and green table represents insertion and deletion signatures (ID)

We explore the mutational pattern presented by the patients of this cohort, C > T changes were the most common, followed by T > C changes (Supplementary Fig. 1). The contribution of single and double base substitution signatures (SBS, DBS), as well as InDel associated mutational signatures from the COSMIC database were estimated for all the patients in this cohort. Among the single substitution signatures, clock-like signature SBS05 was the main contributor for most samples. Within the double base substitution analysis, we observed a high frequency of TG > CA substitutions with a contribution of over 25% (Supplementary Fig. 2). The polymerase epsilon exonuclease domain mutations (DBS03) and the defective DNA mismatch repair system (DBS07) signatures were the most frequently present in this cohort. Finally, insertions and deletion with five or more base pairs were the most common InDel pattern (Supplementary Fig. 3) and the InDel signature associated to the repair of DNA double strand breaks by the non-homologous end joining (NHEJ) and Topoisomerases 2 alpha signature (ID8), was present in all samples, except in PC_16. In Fig. 5, we can observe a summary of the mutation processes of Mexican patients with early-stage prostate adenocarcinoma.

## Discussion

Racial differences based on genomic profiling have been described in African American, White, and Asian PRAD populations. PRAD Hispanic men, on the other hand, are an underrepresented ethnic group with scarce information exploring their genomic profile [32]. This lack of genomic information could hinder the access of Hispanic patients with PRAD to strategies of precision medicine [33]. Hispanics comprise a heterogeneous ethnic group of descendant and individuals from Spanish-speaking countries from North, Central and South Americas [34]. Here we presented a complete genomic profile of a sample of Hispanics—Mexican patients with early-stage PRAD, identifying the genes with driver mutations, the actionable genetic mutations, structural variations, and the mutational processes related to PRAD.

This research specifically focuses on the genomic analysis of prostate cancer within Hispanic populations. This is crucial for advancing our understanding of the disease and addressing health disparities. The fact that only 3% of participants in The Cancer Genome Atlas identify as Hispanic or Latino underscores the urgent need to study this population to reduce prostate cancer disparities [12].

We found *NUP98* mutations in patients with early-stage PRAD. In the study conducted by Liang et al. in 2022, *NUP98* was identified as one of the novel germline predisposing genes to PRAD. From these candidate genes, *NUP98* included, exhibited a significantly higher mutation frequency in the Hong Kong and Shanghai cohorts when compared to controls of East Asian descent [35]. In patients with triple-negative breast cancer, high expression levels of *NUP98,* identified by immunohistochemistry, have been proposed to be a predictor of response to anthracycline-based chemotherapy and poor overall survival [36]. While there is no information on the clinical/predictive utility of NUP98 in patients with PRAD, our results suggest it is one of the main drivers of early stage PRAD in the Hispanic population and, thus, a promising target for further studies exploring its usefulness as a biomarker of prognosis and therapy response.

We also found increased mutations in *CSMD3*, a tumor suppressor gene, where mutations have been shown to lead to the enhancement of tumor cell proliferation and metastasis and, consequently, contributing to adverse clinical manifestations [37]. Mutations in 8q23 affecting *CSMD3* are more frequent in Hispanic population compared with Whites with PRAD, along with upregulated expression linked to chromosomal gains [38]. Amplifications of *CSMD3*, but no mutations, are the most common genomic variation identified in patients with castrate-resistant PRAD in studies from London, UK [39] and Arizona, USA [40]. In our cohort of Mexican patients, we found no amplifications of *CSMD3* and mutations in this gene were the second most common. In hepatocellular carcinoma, mutations of *CSMD3* identified in plasma cell-free tumor DNA were found to be associated with a shorter overall survival [41]. The importance and clinical relevance of *CSMD3* mutations and its relevance to Mexican patients, including prognosis implications, requires further analysis.

Olaparib and Rucaparib are a type of targeted cancer drugs indented to inhibit PARP and used in patients with specific genetic mutations [42]. In PRAD, Olaparib has demonstrated to improve the overall survival of patients with metastatic castration-resistant PRAD [43]. Here we identify that 25% of the Mexican patients comprising this cohort would benefit from the use of a PARP inhibitor due to the presence of mutations in *BRCA2*, *ATM* or *RAD54L.*

When exploring the mutational processes presented in the samples from Mexican patients with early-stage PRAD, we observed a strong influence of SBS05, a mutational signature commonly associated with bladder cancer and the use of tobacco [44]. In this cohort *ARAF* was the most affected gene by SCNA´s, this gene is a member of the RAF kinase family. The *ARAF* expression was unregulated in gallbladder cancer tissues. In a study with west African men with PRAD, SNV’s in 5q31.3 were associated with a Gleason score ≤ 7, like our work with Mexican patients were 18 out of the 20 patients had a Gleason score of 7 and losses in 5q31.3 was the most frequent SCNA [45]. Also, the *ARAF* gene mutation is a rare event in human tumorigenesis but is somatically mutated in human cancers [46].

The study acknowledges the presence of genetic differences in prostate cancer among different racial and ethnic groups. Notably, Black patients exhibit distinct mutations. Understanding the genetic variations within Hispanic populations, including Mexicans, is essential as they may have unique genomic characteristics that influence the disease's behavior and progression. We understand that the sample size of our cohort could limit the statistical power potentially affecting the robustness and generalizability of our findings., however we manage to meat or primary goal and present a complete mutational panorama of early-stage prostate cancer patients, identifying a high frequency of mutations in *NUP93* and *CSMD3*, the influence of mutational processes related to NHEJ DNA repair pathway in early-stage PRAD and the potential benefit of using PARP inhibitors in Mexican patients. To validate and our results a larger cohort of Mexican patients from distinct parts of the country and ethnic backgrounds is needed Future research should aim to include a larger number of patients and involve multiple institutions to enhance the representativeness and statistical power of the findings.

## Conclusions

In this study, we comprehensively analyzed a cohort of 20 Mexican patients with early stage of PRAD, presenting the genomic landscape of this understudied population. Our findings have several key implications for the understanding and management of PRAD in Mexican men. Our data revealed a high prevalence of mutations in the *NUP98* p.R964C mutation in patients with early-stage disease and. Furthermore, our analysis of actionable genetic mutations highlighted the potential for personalized treatment approaches in Mexican PRAD patients. Notably, a subset of patients exhibited mutations in genes associated with PARP inhibitor response, suggesting a therapeutic opportunity for this subgroup. Finally, our work poses insights of the mutational patterns and processes presented by in Mexican men with early-stage PRAD. This study reveals the mutational landscape of early-stage prostate adenocarcinoma in men living in the central area of Mexico. Understanding mutational patterns and actionable mutations in early prostate cancer can inform personalized treatment approaches and reduce the underrepresentation in genomic cancer studies. Further research is needed to explore the implications of these findings.

Even though our study delivers important information about genetic changes in prostate cancer among Hispanic men, its limited sample size restricts its applicability on a global scale. To overcome this limitation, it is essential that future research projects prioritize larger, more diverse participant groups to validate our findings and investigate the genetic aspects of prostate cancer more thoroughly. These efforts will be vital for enhancing patient care and creating personalized treatments.

### Supplementary Information

Supplementary Material 1.

## Data Availability

The data presented in this study are available upon request to the corresponding author if you want to partner with or contribute to the project. The data are not publicly available due to INCan-Mx policy for genomic data of patients.

## Abbreviations

BAM: Binary Alignment Map
BWA-MEM: Burrows-Wheeler Aligner
CGI: Cancer Genome Interpreter
COSMIC: Catalogue of Somatic Mutations in Cancer
DBS: Double base substitutions
DGV: Database of genomic variants
DNA: Deoxyribonucleic acid
ER: Enrichment ratio
FFPE: Formalin-fixed paraffin-embedded
GATK: The Genome Analysis Toolkit
ID: Insertion and deletion
INCAN: Instituto Nacional de Cancerología
NHEJ: Non-homologous DNA end joining
PARP: Poly (ADP-ribose) polymerases
PRAD: Prostate adenocarcinoma
SBS: Single-base substitutions
SCNA: Somatic copy number alterations
SD: Standard deviation
SNV: Single nucleotide variants
WES: Whole exome sequencing
