# Supplementary material for: Genomic landscape of early-stage prostate adenocarcinoma in Mexican patients: an exploratory study
Source: Discov Oncol. 2024 Aug 28;15:378. doi: 10.1007/s12672-024-01199-3 (PMC11358564; doi:10.1007/s12672-024-01199-3)
Supplement: Supplementary file 1 — Supplementary Material 1. [file 12672_2024_1199_MOESM1_ESM.pdf]

Genomic Landscape of Early-Stage Prostate Adenocarcinoma in Mexican patients: An exploratory study

Supplementary Material

Figure supplementary 1: Mutational Profile SBS

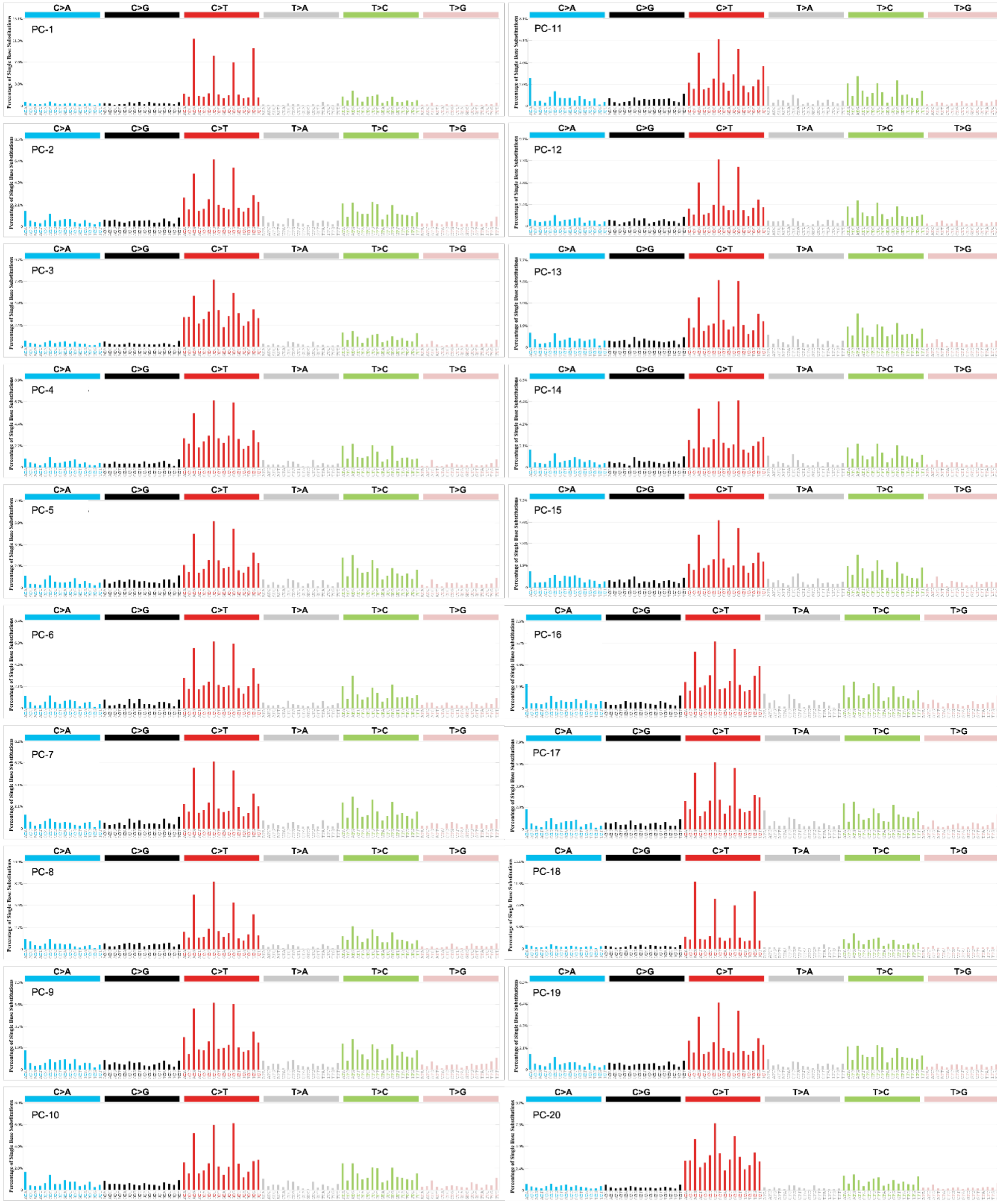

Supplementary Figure 1. Single Base Substitution (SBS) mutational profile. Bar graph depicting the SBS profile by each patient in a 96-base context.

Figure supplementary 2: Mutational Profile DBS

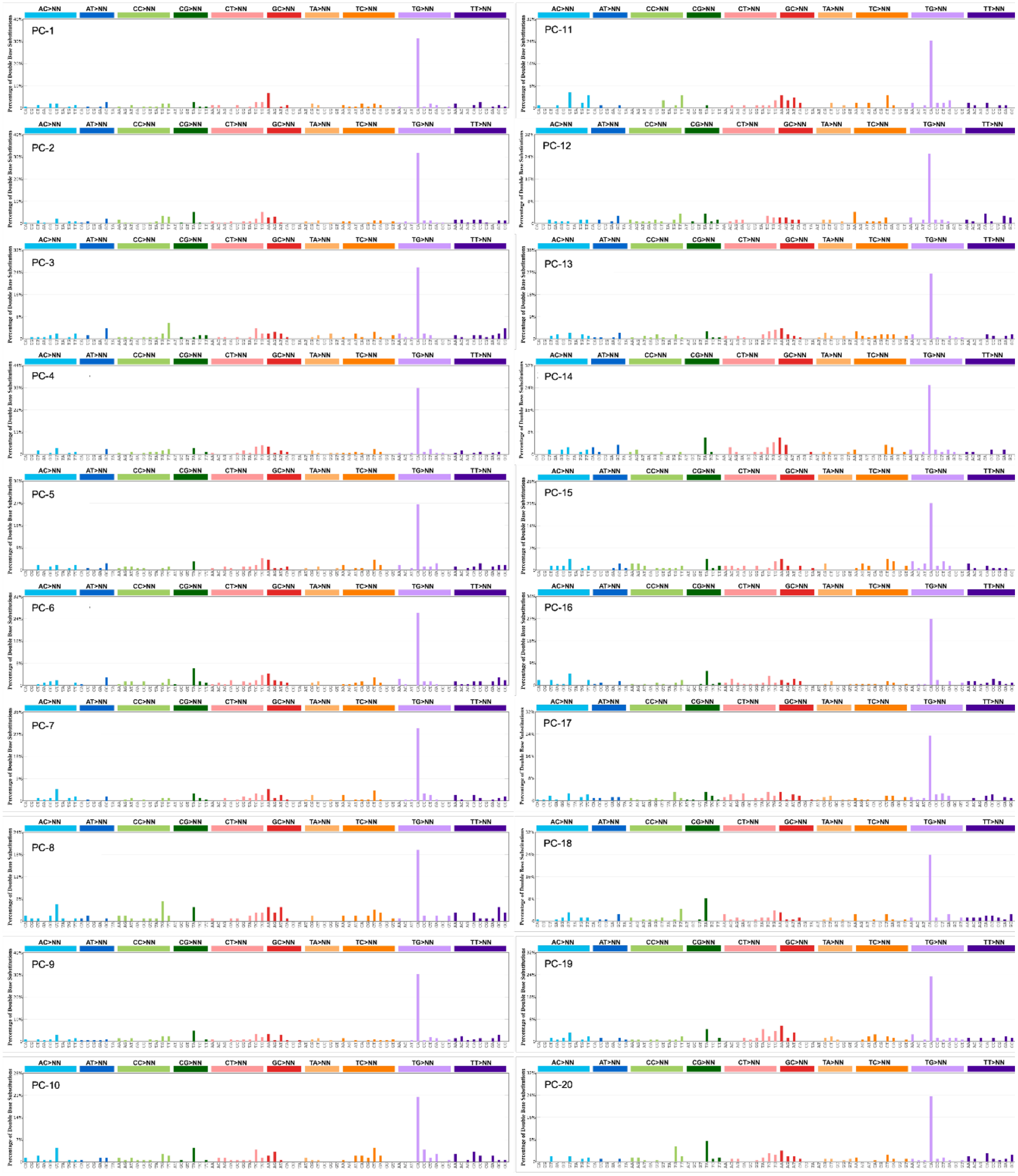

Supplementary Figure 2. Doble Base Substitution (DBS) mutational profile. Bar graph depicting the DBS profile by each patient

Figure supplementary 3: Mutational Profile InDels

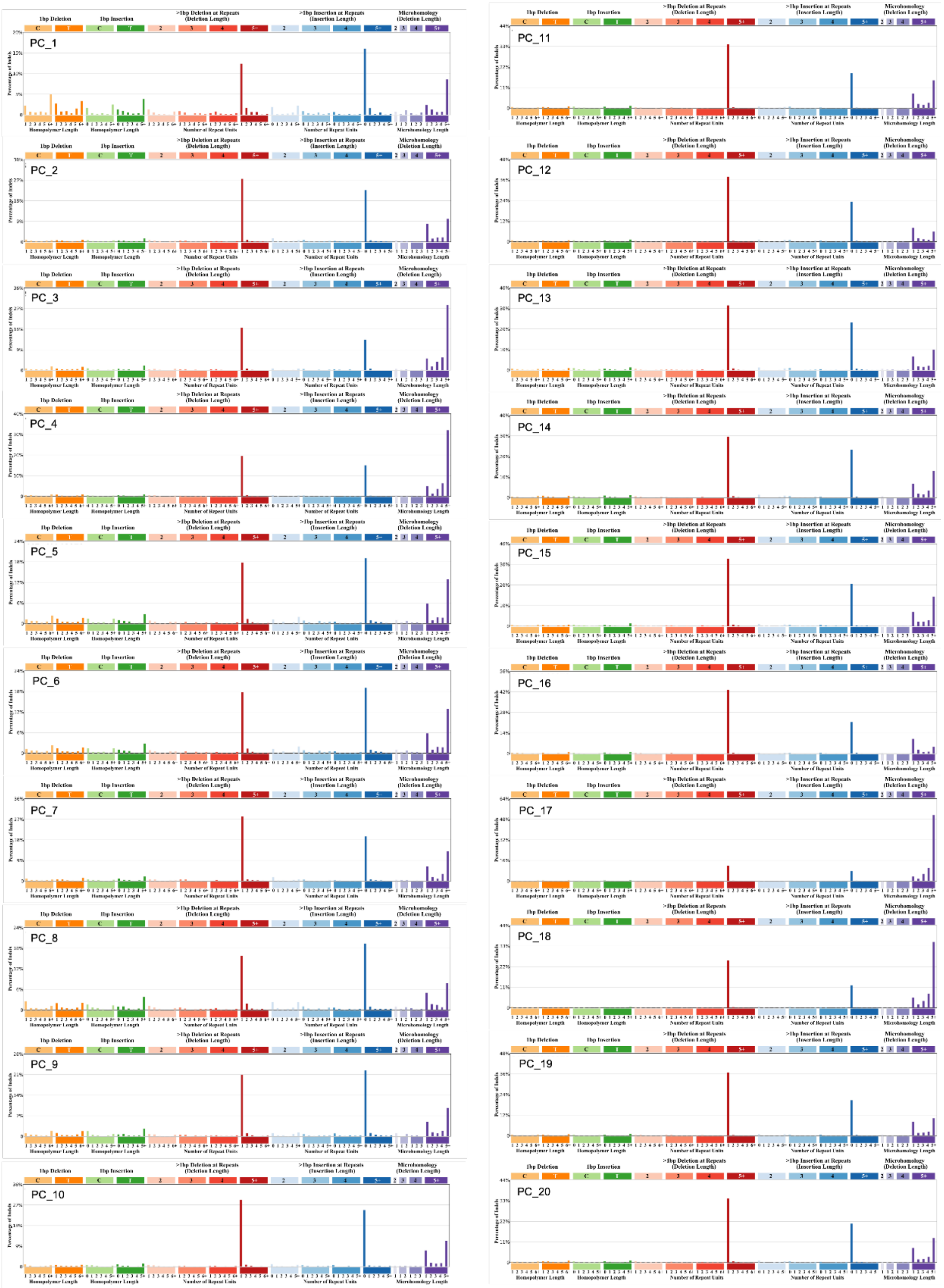

Supplementary Figure 3. Mutational profile of small insertions and deletions (ID). Bar graph depicting the ID profile by each patient
